# Anti-CD7 antibody and immunotoxin treatment of human CD7^+^T-cell leukaemia is significantly less effective in NOD/LtSz-scid mice than in CB.17 scid mice

**DOI:** 10.1054/bjoc.2000.1565

**Published:** 2000-12

**Authors:** D J Flavell, S L Warnes, A L Noss, S U Flavell

**Affiliations:** The Simon Flavell Leukaemia Research Unit, Division of Cancer Sciences, University of Southampton School of Medicine, Southampton General Hospital, Southampton, Hampshire, SO16 6YD, UK

## Abstract

Groups of 8 to ten SCID (CB.17 *scid/scid*) or NOD/SCID (NOD/LtSz- *scid/scid*) mice were injected i.v. with two million human HSB-2 T-ALL cells on day 1 (SCID-HSB-2 and NOD/SCID-HSB-2 mice) and treated later with 3 i.v. 10 μg doses of the anti-CD7 antibody HB2 on days 7, 9 and 11 or with a single 10 μg dose of HB2-SAPORIN or a 7.4 μg dose of HB2-F(ab)_2_-SAPORIN immunotoxin (IT) on day 7. Treatment of SCID-HSB-2 mice with HB2-SAPORIN led to a significant prolongation in the time to development of signs and symptoms of disease compared with PBS sham-treated controls with 80% of animals surviving disease-free. In contrast treatment with HB2-F(ab)_2_-SAPORIN was significantly less effective in SCID-HSB-2 mice with 80% of animals in this treatment group developing leukaemia over the course of the study. HB2 antibody treatment of SCID-HSB-2 mice also led to a significant prolongation in time to leukaemia development compared with sham-treated controls with 37% of animals in this treatment group disease-free at termination of the study. In contrast HB2 antibody treatment of NOD/SCID-HSB-2 mice had no therapeutic effect in these animals and the therapeutic effectiveness of both HB2-SAPORIN and HB2-F(ab)_2_-SAPORIN ITs was similar and significantly reduced compared to the effect observed in SCID-HSB-2 mice. It was initially thought that the lack of therapeutic effect of antibody and IT in NOD-SCID-HSB-2 mice might relate to their putative lack of NK cells but flow cytometric and functional studies with NOD-SCID mouse splenocytes revealed that these animals do have some functional NK cells though fewer in number and possibly lower in functionality than those of SCID mice. We reason that the complete lack of therapeutic effect of HB2 antibody and the reduced effect of HB2-SAPORIN in NOD/SCID mice is due to the reduced cytolytic activity of NOD/SCID NK cells which is probably below a certain critical threshold value in these animals. We conclude from this that immunotherapeutics like HB2-SAPORIN would be more accurately assessed for intrinsic potency in NOD/SCID mice where the effects of NK cell and possibly other non-adaptive immune mechanisms would not have a significant influence. © 2000 Cancer Research Campaign http://www.bjcancer.com


*British Journal of Cancer* (2000) **83**(12), 1755–1761
© 2000 Cancer Research Campaign
doi: 10.1054/ bjoc.2000.1565, available online at http://www.idealibrary.com on IDEAL®

### Anti-CD7 antibody and immunotoxin treatment of human CD7⁺ T-cell leukaemia is significantly less effective in NOD/LtSz-scid mice than in CB.17 scid mice

**DJ Flavell, SL Warnes, AL Noss and SU Flavell**

The Simon Flavell Leukaemia Research Unit, Division of Cancer Sciences, University of Southampton School of Medicine, Southampton General Hospital, Southampton, Hampshire SO16 6YD, UK

**Summary** Groups of 8 to ten SCID (CB.17 *scid/scid*) or NOD/SCID (NOD/LtSz-*scid/scid*) mice were injected i.v. with two million human HSB-2 T-ALL cells on day 1 (SCID-HSB-2 and NOD/SCID-HSB-2 mice) and treated later with 3 i.v. 10 μg doses of the anti-CD7 antibody HB2 on days 7, 9 and 11 or with a single 10 μg dose of HB2-SAPORIN or a 7.4 μg dose of HB2-F(ab)$_2$-SAPORIN immunotoxin (IT) on day 7. Treatment of SCID-HSB-2 mice with HB2-SAPORIN led to a significant prolongation in the time to development of signs and symptoms of disease compared with PBS sham-treated controls with 80% of animals surviving disease-free. In contrast treatment with HB2-F(ab)$_2$-SAPORIN was significantly less effective in SCID-HSB-2 mice with 80% of animals in this treatment group developing leukaemia over the course of the study. HB2 antibody treatment of SCID-HSB-2 mice also led to a significant prolongation in time to leukaemia development compared with sham-treated controls with 37% of animals in this treatment group disease-free at termination of the study. In contrast HB2 antibody treatment of NOD/SCID-HSB-2 mice had no therapeutic effect in these animals and the therapeutic effectiveness of both HB2-SAPORIN and HB2-F(ab)$_2$-SAPORIN ITs was similar and significantly reduced compared to the effect observed in SCID-HSB-2 mice. It was initially thought that the lack of therapeutic effect of antibody and IT in NOD-SCID-HSB-2 mice might relate to their putative lack of NK cells but flow cytometric and functional studies with NOD-SCID mouse splenocytes revealed that these animals do have some functional NK cells though fewer in number and possibly lower in functionality than those of SCID mice. We reason that the complete lack of therapeutic effect of HB2 antibody and the reduced effect of HB2-SAPORIN in NOD/SCID mice is due to the reduced cytolytic activity of NOD/SCID NK cells which is probably below a certain critical threshold value in these animals. We conclude from this that immunotherapeutics like HB2-SAPORIN would be more accurately assessed for intrinsic potency in NOD/SCID mice where the effects of NK cell and possibly other non-adaptive immune mechanisms would not have a significant influence. © 2000 Cancer Research Campaign http://www.bjcancer.com

The discovery of the severe combined immune deficient (SCID) mouse by Bosma and co-workers (Bosma et al, 1983) opened up avenues that allowed researchers to exploit this strain of mouse for the xenografting of human tumours and normal tissues in ways that had not previously been possible. The SCID mouse proved of particular value to workers interested in the leukaemias and in haematopoiesis, allowing for early engraftment experiments initially with cell lines (Kamel-Reid et al, 1989) and later with primary haematological malignant (Cesano et al, 1991; Kamel-Reid et al, 1991) and non-malignant cells (Lapidot et al, 1992; Kollmann et al, 1994). The relative ease with which SCID mice accept xenografts can be attributed to their virtual total lack of functional B- and T-cells. This is due to a defect in their recombinase system that prevents immunoglobulin and T-cell receptor (TCR) gene rearrangements that would normally drive the diversity of the antigen recognition repertoire in normal mice (Kotloff et al, 1993). Subsequently progenitor B- and T-cells fail to develop to a fully functional mature state in SCID mice (Custer et al, 1985) and instead undergo apoptosis early in their life history. SCID mice do however possess a virtually fully functional non-adaptive immune system with normal levels of tissue macrophages, NK cells, serum complement activity and normal myelopoiesis (Dorshkind et al, 1984, 1985). The presence of functional NK cells and tissue macrophages has in some instances created a barrier to successful engraftment with both normal and malignant primary haematological material. This problem has been at least partially overcome in the past by depleting SCID mice of their NK cells by treatment prior to xenografting with non-lethal doses of total body radiation, anti-asialo GM1 antibody (Shpitz et al, 1994) or cytotoxic drugs such as cyclophosphamide. NK cells are however only transiently eliminated by such methods and this makes the SCID mouse of limited value in long-term engraftment studies where the reappearance of NK cells might likely compromise the biological behaviour of the graft.

Non-Obese Diabetic mice (NOD/Lt strain) have a significant deficit in their non-adaptive immunity with reductions in NK cell functionality (Kataoka et al, 1983; Serreze and Leiter, 1988), an absence of circulating complement components (Baxter and Cooke, 1993) and functional defects in antigen presenting cells (APC) (Serreze et al, 1993). NOD/Lt mice develop a high incidence of autoimmune (type 1) insulin-dependent diabetes mellitus (IDDM) mediated by auto reactive T-cells (Christianson et al, 1993). This led Shultz and co-workers (Shultz et al, 1995) to backcross the *scid* mutation onto the NOD/Lt strain background, resulting in an immunodeficient mouse (NOD/LtSz-*scid/scid*) with multiple defects in adaptive as well as non-adaptive

*Received 12 June 2000*
*Revised 25 August 2000*
*Accepted 5 October 2000*

*Correspondence to:* DJ Flavell immunity, which for the purposes of this paper are termed NOD/SCID mice. Moreover, the profound B- and T-cell deficiency acquired through the *scid* mutation means that NOD/SCID mice do not develop IDDM but they do have a high incidence for developing spontaneous thymomas (Prochazka et al, 1992) giving them a life expectancy of 12 months or less. The profound immunodeficient state of NOD/SCID mice has resulted in better and more robust engraftment levels with normal and malignant haematological material (Wang et al, 1995) that has allowed for a quantitative evaluation of leukaemic stem cell populations in primary patient derived samples (Dick, 1996) and the establishment of intact human haematopoietic systems (Bhatia et al, 1997).

SCID mice have in the past provided a useful tool for the preclinical evaluation of novel antibody-based therapies for human acute leukaemias and lymphomas. Nearly all of these previous studies have been based upon cell line models, which may not accurately recapitulate the disease process and behaviour in humans (Flavell, 1996). A major advance in this area would be to develop the capability of engrafting a sufficiently large enough cohort of animals with human leukaemia cells from one individual patient in order to undertake therapy studies with sufficient statistical power. In this context several workers have recently reported the several-fold expansion of human T-cell leukaemia (Yu et al, 1999) and myeloma cells (Pilarski et al, 2000) in preconditioned NOD/SCID. As a preliminary undertaking to progressing the idea of developing in vivo therapy models for individual patients for the preclinical evaluation of antibody-based therapeutics we wanted to ensure that such therapy studies were feasible in NOD/SCID mice utilizing an existing cell line model of human T-ALL, which has been well defined in the SCID mouse. Our strategy has been to compare the therapeutic efficacy of the murine anti-CD7 $IgG_1$ antibody HB2 and the immunotoxin HB2-SAPORIN in SCID and NOD/SCID mice engrafted with the human $CD7^+$ T-ALL cell line HSB-2. We clearly demonstrate that the therapeutic effectiveness of HB2 antibody is significant in SCID mice but is non-existent in NOD/SCID mice and also show that HB2-SAPORIN IT treatment is significantly reduced in NOD/SCID when compared with the outcome obtained in SCID mice. These current experimental findings taken together with our previous observations (Flavell et al, 1998) lead us to propose that the NOD/SCID mouse model of HSB-2 human leukaemia more accurately reflects the intrinsic in vivo therapeutic effectiveness of HB2-SAPORIN treatment which unlike the SCID mouse model is not influenced by the presence of NK cells which we have previously shown can have significant effects on the in vivo therapeutic potency of IT treatment.

### MATERIALS AND METHODS

### SCID and NOD/SCID mice

Pathogen free CB.17 *scid/scid* (SCID) mice of both sexes were produced from our own breeding colony. NOD/LtSz-*scid/scid* mice (NOD/SCID) were also provided from our own breeding colony with breeding pairs kindly supplied by Dr Finbarr Cotter (Institute of Child Health, London, UK). All animals were maintained in filter top micro isolator cages and provided with sterile water and food ad libitum under conditions complying with British Government Home Office regulations. All manipulations of experimental animals were conducted in a laminar flow hood using strictly controlled procedures adhering to the UKCCCG Guidelines for the Welfare of Animals in Experimental Neoplasia to minimize stress or suffering (Workman et al, 1998).

### HSB-2 human T-ALL cell line and YAC-1 murine lymphoma cell line

The $CD7^+$ human cell line HSB-2 (Adams et al, 1970) and the murine lymphoma cell line YAC-1 (Cikes et al, 1973) were maintained in the logarithmic phase of growth in RPMI 1640 medium containing 10% fetal calf serum and supplements of 2 mM of sodium pyruvate and glutamine (R10 medium) at 37˚C under a humidified atmosphere of 5% $CO_2$.

### Antibodies

The rat anti-mouse antibodies AT37 (anti-CD2), 6D5 (anti-CD19) and F4/80 (anti-macrophage) and the mouse anti-mouse NK cell antibody NK1-1 directly conjugated to phycoerythrin were obtained from Serotec Ltd (Kidlington, UK). The rat antibody 2.4G2 directed against mouse FcγRII/III (anti-CD16/32) was obtained from PharMingen (San Diego, CA). The murine hybridoma cell line HB2 producing an $IgG_1$ antibody directed against the human CD7 molecule was obtained from the American Tissue Culture Collection (Bethesda, MD). Bulk HB2 antibody was produced in vitro from this hybridoma on a Cellex Accusyst R hollow fibre bioreactor system (Cellex, Minneapolis, MN, USA). $F(ab')_2$ fragments of HB2 antibody were produced by pepsin digestion of native HB2 antibody employing an ImmunoPure $F(ab')_2$ kit from Pierce, (Rockford, IL, USA) following the manufacturers instructions. $F(ab')_2$ HB2 antibody produced in this way gave a single band of 110 kD molecular weight on SDS-PAGE analysis under non-reducing conditions and was wholly free of detectable contaminating Fc.

### Immunotoxin construction

The immunotoxins HB2-SAPORIN and HB2-$F(ab')_2$-SAPORIN were constructed by conjugating molar equivalent amounts of HB2 antibody or its $F(ab')_2$ fragment to saporin using the heterobifunctional cross linking reagent N-succinimidyl 3-(2-pyridyldithio)propionate (SPDP) as described previously (Thorpe et al, 1985). Only IT containing 2 saporin moieties per IT molecule were used in these studies because of their well defined characteristics and potency as described by us previously (Flavell et al, 1997). The purity of immunotoxins was confirmed by SDS-PAGE and were then dialysed into PBS pH7.2, sterilized by passage through a 0.2 μm filter and stored deep frozen in 100 μg aliquots at −80˚C.

### Chromium release assay

Details of chromium release assays used to determine the lytic capability of SCID and NOD/SCID splenocytes for HSB-2 and YAC-1 cells both constitutively and as effectors in antibody-dependent cellular cytotoxicity (ADCC) have been fully described previously (Flavell et al, 1998).

### Flow cytometry

Expression of the various antigen markers on SCID and NOD/SCID splenocytes was determined on a Coulter Epics XL flow

 © 2000 Cancer Research Campaign cytometer following indirect or direct staining of splenocytes with the appropriate antibody as described (Flavell et al, 1998).

### In vivo experiments

On day one of study, groups of 10 SCID or NOD/SCID mice were injected into the tail vein with two million HSB-2 cells in a 200 μl volume of R10 medium (termed SCID-HSB-2 or NOD/SCID-HSB-2 animals). We have described the growth and dissemination of the human T-ALL cell line HSB-2 in SCID mice in detail previously (Morland et al, 1994). We have observed that the pattern of disease development and progression is virtually identical in NOD/SCID mice (unpublished observations). The appropriate groups of SCID-HSB-2 or NOD/SCID-HSB-2 mice were treated i.v. with three 10 μg doses of HB2 antibody given on days 7, 9 and 11 or sham treated with three doses of PBS given at the same time points. Other groups of SCID-HSB-2 or NOD/SCID-HSB-2 animals received a single 10 μg i.v. dose of HB2-SAPORIN or with a molar equivalent amount (7.4 μg) of HB2-F(ab)$_2$-SAPORIN on day 7 or were sham treated with a single i.v. injection of 200 μl PBS on day 7. All animals were monitored twice daily for the duration of the study and strictly defined endpoints were enforced as per recommendations under the UKCCCR guidelines (Workman et al, 1998) to ensure that no animal suffered undue or unnecessary stress or pain during the course of the study. Thus, animals showing any identifiable early signs of being unwell were killed humanely and full post-mortem examinations conducted to confirm the presence of tumour.

### Statistical analysis

Log-Rank analysis (Peto's method) using the SOLO statistics software package (BMDP Statistical Software. Los Angeles, CA, USA) was used to determine if there were any significant differences between the various therapy groups. *P* values of 0.05 or less were considered as statistically significant in these studies.

### RESULTS

### Immunophenotypic analysis of SCID and NOD/SCID splenocyte populations

Comparative immunophenotypic analysis of splenocytes taken from SCID and NOD/SCID mice was undertaken by single colour flow cytometry and revealed the differences shown in Table 1. 32% of the splenic population of SCID mice were positive for the NK cell marker NK1.1 compared with only 20% of NOD/SCID splenocytes. Similarly there were fewer NOD/SCID splenocytes expressing FcgRII/RIII compared with SCID splenocytes (22% versus 37%, respectively) and also fewer macrophages identified by the F4/80 antibody in NOD/SCID spleens compared with SCID spleens (12% versus 17%). There were also marginally fewer CD2 expressing splenocytes in NOD/SCID mice but similar numbers of CD19 expressing cells.

**Table 1** Immunophenotypic composition of splenocytes from CB.17 SCID and NOD/SCID mice as determined by flow cytometry

| Surface antigen | % positive splenocytes | |
|---|---|---|
| | SCID | NOD/SCID |
| NK 1.1 | 32 | 20 |
| F4/80 | 17 | 12 |
| CD16/32 | 37 | 22 |
| CD2 | 5 | 2 |
| CD19 | 2 | 2 |

### Functional characteristics of SCID and NOD/SCID splenocytes

*Constitutive cytotoxicity for YAC-1 and HSB-2 cells*

Splenocytes taken from SCID and NOD/SCID mice that had been treated 24 h previously with 100 μg poly IC i.v. were tested for

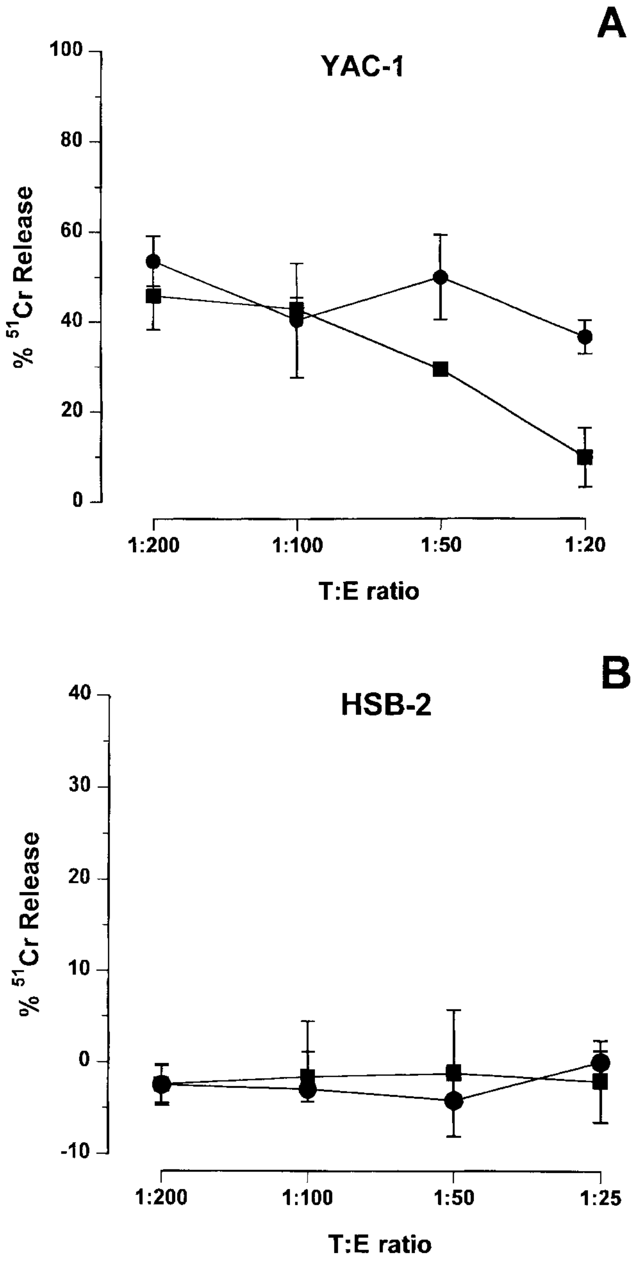

**Figure 1** Lysis of (**A**) target YAC-1 cells or (**B**) HSB-2 cells expressed as a percentage of $^{51}$Cr release following incubation with either CB.17 SCID (●) or NOD/SCID (■) effector splenocytes at target:effector ratios ranging from 1:200 to 1:20. Bars, SD

© 2000 Cancer Research Campaign

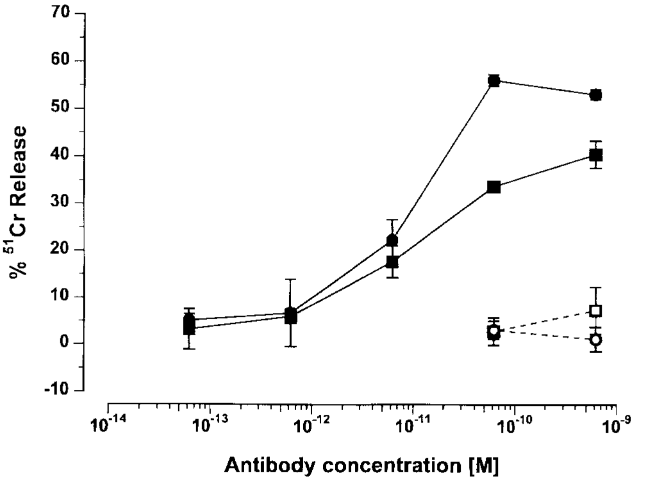

**Figure 2** Lysis of target HSB-2 cells by ADCC elicited by SCID (●) or NOD/SCID mouse (■) effector splenocytes at a 100:1 E:T ratio in the presence of increasing concentrations of the anti-CD7 antibody HB2. Lysis of target HSB-2 cells in the presence of the irrelevant anti-CD19 isotype matched control antibody BU-12 with CB.17 SCID (○) or NOD/SCID (□) splenocytes is also shown. Bars, SD

their ability to lyse target YAC-1 or HSB-2 cells in a chromium release assay at target to effector (T:E) ratios ranging from 1:200 to 1:20. Figure 1A shows that at T:E ratios of 1:200 and 1:100 SCID and NOD/SCID splenocytes performed comparably, eliciting a similar degree of lysis of YAC-1 cells. However, at T:E ratios of 1:50 and 1:20 NOD/SCID splenocytes declined in their ability to lyse YAC-1 cells while SCID splenocytes maintained their capacity at a level similar to that seen at the higher T:E ratios. Neither SCID or NOD/SCID splenocytes were capable of lysing HSB-2 cells at any of the T:E ratios studied (Figure 1B).

### *Antibody-dependent cellular cytotoxicity (ADCC)*

Both SCID and NOD/SCID splenocytes were tested for their ability to lyse target HSB-2 cells in an antibody-dependent cellular cytotoxicity (ADCC) assay at a T:E ratio of 1:100 in the presence of increasing concentrations of the anti-CD7 antibody HB2. The results of this study are shown in Figure 2. The greatest degree of target cell lysis was achieved with SCID mouse splenocytes at the two highest antibody concentrations of 660 and 66 pM being 55% and 52%, respectively compared with 31% and 37% lysis obtained with NOD/SCID splenocytes at these same antibody concentrations. In the same assay the irrelevant isotype matched control antibody BU-12 (anti-CD19) used at the two highest concentrations did not cause lysis of HSB-2 cells when either SCID or NOD/SCID splenocytes were employed.

### Therapeutic effects of murine anti-CD7 antibody treatment in SCID-HSB-2 and NOD/SCID-HSB-2 mice

SCID mice injected i.v with two million HSB-2 cells on day 1 and treated with three 10 μg i.v. doses of HB2 antibody on days 7, 9 and 11 showed a significant ($P = 0.0135$) prolongation in the time taken to develop symptoms compared with PBS sham-treated SCID mice, with 37% of these animals alive and disease-free at elective termination of the experiment at 135 days (Figure 3A). In contrast HB2 antibody showed no significant therapeutic effect ($P = 0.243$) in identically treated NOD/SCID mice, with antibody-treated animals developing symptoms at virtually the same rate as PBS sham-treated

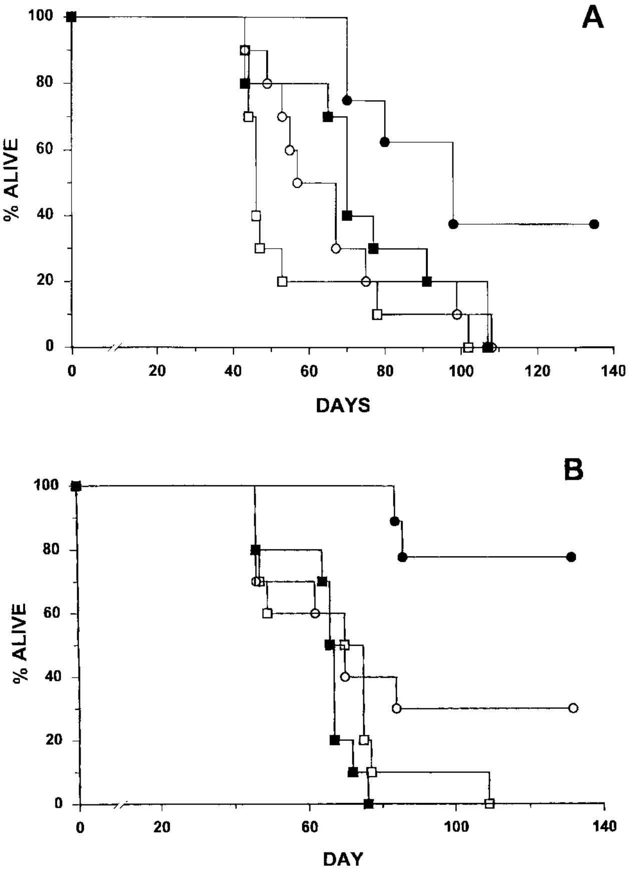

**Figure 3** Kaplan-Meyer plots for groups of SCID (closed symbols) or NOD/SCID mice (open symbols) injected i.v. on day 1 with two million HSB-2 cells and treated with (**A**) three 10 μg i.v. injections of HB2 antibody given on days 7, 9 and 11 (●, ○) or sham treated at the same times with 200 μl of PBS (■, □) or (**B**) treated with a single 10 μg i.v. injection of HB2-SAPORIN on day 7 (●, ○) or sham treated at the same time with 200 μl PBS (■, □)

NOD/SCID control animals and with all animals in the antibody-treated group dead by 102 days (Figure 3A). In this instance NOD/SCID PBS sham-treated controls succumbed to disease at a slightly faster rate overall than the equivalent SCID control animals, though the difference between them was not significant (Figure 3A).

### Therapeutic effects of HB2-SAPORIN and HB2-F(ab)$_2$-SAPORIN in SCID-HSB-2 and NOD/SCID-HSB-2 mice

To determine the therapeutic effect of the anti-CD7 HB2-SAPORIN IT in SCID-HSB-2 and NOD/SCID-HSB-2 mice we injected a single 10 μg i.v. dose of HB2-SAPORIN IT into mice 7 days after i.v. challenge with two million HSB-2 cells. The IT had a highly significant therapeutic effect ($P = 0.0002$) in SCID-HSB-2 mice compared with PBS sham-treated SCID control animals, with 80% of the IT treated animals alive and disease-free for the 135 day duration of the experiment (Figure 3B). In identically treated NOD/SCID mice the IT was therapeutically ineffective in the majority of animals, with 70% of these animals developing signs and symptoms of leukaemia growth at the same rate as the PBS sham-treated control animals (Figure 3B) but with an eventual 30% of animals disease-free at elective termination of the experiment. However, despite this 30% survival rate in the IT-treated NOD/SCID group

 © 2000 Cancer Research Campaign

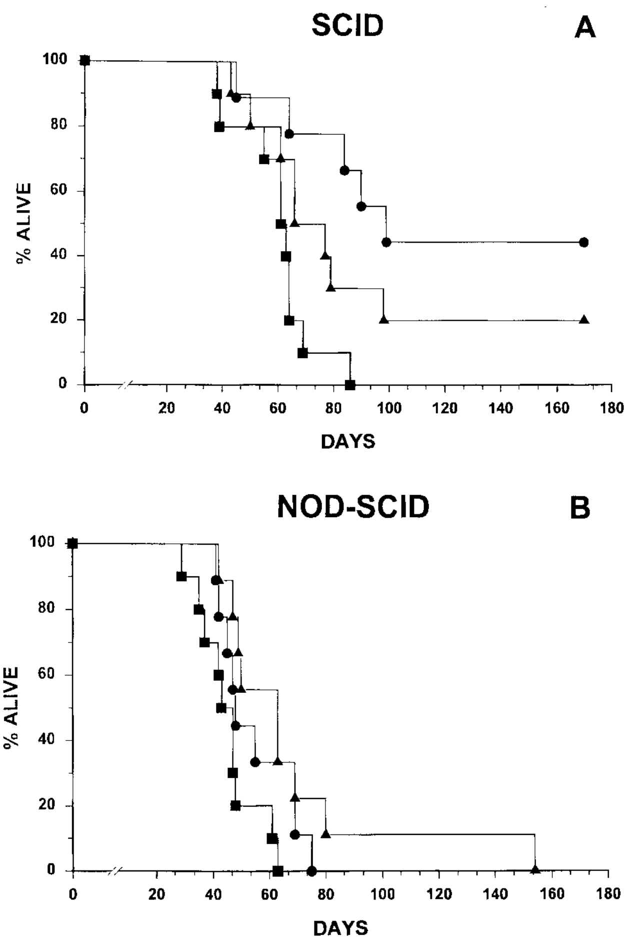

**Figure 4** Kaplan-Meyer plots for groups of (**A**) SCID mice and (**B**) NOD/SCID mice treated with a single i.v. injection of 10 μg HB2-SAPORIN IT (●), 7.4 μg HB2-F(ab)$_2$-SAPORIN (▲) or sham treated at the same time with 200 μl PBS (■)

(compared with a zero survival rate in the NOD/SCID controls) the therapeutic effect was not found to be significant by log-rank analysis ($P$ = 0.296). This was probably because the rapid rate at which the majority of animals developed disease early in the experiment skewed the statistical test. In this particular instance SCID and NOD/SCID PBS sham-treated animals died at a very similar rate and thus there was no significant difference between them.

We also conducted an experiment to compare the therapeutic effects of HB2-SAPORIN and HB2-F(ab)$_2$-SAPORIN immunotoxins in SCID-HSB-2 and NOD/SCID-HSB-2 mice and the results we obtained are shown in Figure 4. In SCID-HSB-2 mice the HB2-F(ab)$_2$-SAPORIN IT performed significantly less well therapeutically than the HB2-SAPORIN IT constructed with intact HB2 antibody (Figure 4A). In this instance the HB2-F(ab)$_2$-SAPORIN-treated animals developed signs and symptoms of leukaemia at a more rapid rate than animals treated with HB2-SAPORIN and moreover only 20% of these animals were alive and leukaemia-free upon elective termination of the experiment at 170 days, in contrast to the 43% of animals treated with HB2-SAPORIN IT. Figure 3B shows that there was no significant difference in the therapeutic performance of either IT in NOD/SCID-HSB-2 mice. In this instance both HB2-SAPORIN and HB2-F(ab)$_2$-SAPORIN were almost completely ineffective, with animals from both IT therapy groups developing signs and symptoms of leukaemia only at a slightly slower rate than the PBS control group. There was one single animal from within the HB2-F(ab)$_2$-SAPORIN treatment group that only developed signs and symptoms of HSB-2 leukaemia growth after 151 days, otherwise all other animals developed disease at a similar rate to HB2-SAPORIN-treated animals. The differences between the HB2-SAPORIN and HB2-F(ab)$_2$-SAPORIN-treated animals and PBS sham-treated controls were not significant. Therefore in this particular experiment the lack of therapeutic effect of HB2-SAPORIN IT in NOD/SCID-HSB-2 mice was even more pronounced than in the previously described experiment illustrated in Figure 2B.

### DISCUSSION

The two major findings to emerge from this study are (a) that the therapy of NOD/SCID mice bearing the human CD7$^+$ T-cell acute leukaemia cell line HSB-2 with the murine IgG1 antibody HB2 directed against the human CD7 molecule is therapeutically totally ineffective in contrast to the significant therapeutic effect that is achieved with this antibody in SCID mice and (b) that the therapeutic effectiveness of the anti-CD7 IT HB2-SAPORIN is significantly reduced in NOD/SCID-HSB-2 mice compared with that obtained in SCID-HSB-2 mice. The in vivo experiments showing this, have been repeated on three separate occasions with similar results each time and two of these have been reported here. On each occasion the therapeutic effect of HB2 antibody or HB2-SAPORIN IT has been found to be significantly reduced in NOD/SCID-HSB-2 mice compared with SCID-HSB-2 mice. In one experiment reported here there was virtually no observable therapeutic effect of HB2-SAPORIN in NOD/SCID-HSB-2 mice (see Figure 4B) and in the other two studies, one of which is reported here (see Figure 3B), there was a therapeutic effect in a proportion of the NOD/SCID-HSB-2 mice but overall this effect was significantly reduced. We have previously shown that the antibody-mediated anti-leukaemia effect that is exerted by HB2 antibody in SCID-HSB-2 mice is very likely mediated by SCID mouse NK cells (Flavell et al, 1998) which we and others have shown to be functionally present in SCID mice (Dorshkind et al, 1985; Flavell et al, 1998). We were also able to show in the present study that NOD/SCID mice possess functionally intact NK cell-like activities that were capable of lysing target YAC-1 cells and of also participating in ADCC against antibody coated target HSB-2 cells. However, it would appear that there are fewer FcγRII/RIII and NK1.1$^+$ NK cells present in the spleens of NOD/SCID mice. Furthermore our study also suggests that NOD/SCID NK cells are probably functionally less active than the equivalent SCID mouse cell counterparts. Thus we were able to demonstrate in the present study a diminishing lytic capability of NOD/SCID mouse splenocytes for YAC-1 target cells at lower T:E ratios that was not paralleled by SCID splenocytes when used at the same ratios and furthermore a reduced lytic capacity of NOD/SCID splenocytes against HSB-2 cells in an ADCC assay compared with that seen with SCID splenocytes. Despite this, it is quite surprising that splenocytes from NOD/SCID mice, which do appear to possess some in vitro lytic NK cell-like activities, albeit at reduced levels, fail to elicit an effective in vivo ADCC anti-leukaemia response with HB2 antibody. One possibility is that this is because the functional quality of the relevant NOD/SCID splenocyte population is below the threshold value to be effective in vivo. An alternative explanation might be that the extra-splenic tissue distribution of functional NK cells is sparse in NOD/SCID mice and/or that the migration of

© 2000 Cancer Research Campaign

NK cells into tissues is in some way impaired in these animals. The latter would prevent NK cells coming into direct contact with HSB-2 cells in extra-splenic tissue locations and thus prevent the engagement of NK cell surface FcγRIII with the Fc domain of antibody coating the target leukaemia cell, a step that is an absolute requirement for cellular cytotoxicity. This study has not attempted to examine the distribution of NK1.1 cells in other NOD/SCID tissues other than spleen and it is therefore not possible to draw any firm conclusions regarding these various possibilities at this stage, though it is the opinion of the authors that it is the impairment in NOD/SCID NK cell lytic function that more likely accounts for the apparent lack of in vivo directed ADCC against HSB-2 cells.

We also demonstrated in the present study that the anti-CD7 IT HB2-SAPORIN was therapeutically significantly less effective in vivo against HSB-2 cells xenografted into NOD/SCID mice in comparison to the therapeutic effects obtained in SCID mice. We observed in one experiment that 70% of NOD/SCID-HSB-2 mice treated with IT developed signs and symptoms of leukaemia cell growth at virtually the same rate as PBS sham-treated controls, and achieved ultimately only a 30% disease-free survival rate. This compares with an 80% survival rate in equivalent SCID mice treated with the same IT. This was even more pronounced in a second experiment where HB2-SAPORIN was almost completely ineffective, giving only a very modest increase in the time taken to develop symptoms compared with PBS sham-treated controls and with all animals in this treatment group eventually showing demonstrable leukaemia cell growth. The HB2-F(ab)$_2$-SAPORIN IT. which is incapable of recruiting NK cells due to its lack of an Fc domain, performed in a near identical fashion to HB2-SAPORIN. These findings in NOD/SCID mice are remarkably similar to the observed outcome when standard SCID mice bearing HSB-2 leukaemia are depleted of their NK cells with anti-asialo GM1 antibody and then treated with HB2-SAPORIN IT (Flavell et al, 1998). Here the therapeutic effect of HB2-SAPORIN was significantly reduced in NK cell-depleted animals in comparison to NK cell-intact animals. Just as in NOD/SCID-HSB-2 mice treated with the IT HB2-SAPORIN in the present study, the majority of SCID-HSB-2 mice depleted of their NK cells with anti-asialo GM1 antibody also died at a similar rate as sham-treated controls (Flavell et al, 1998). This allowed us to draw the conclusion that ADCC mediated by NK or NK-like cells contributed additively to the overall therapeutic effect of HB2-SAPORIN in the SCID-HSB-2 model. The present study would seem to provide further support for this hypothesis, that is to say that it is the absence of effective in vivo ADCC in NOD/SCID-HSB-2 mice that reduces the therapeutic effect of the IT HB2-SAPORIN. The cautionary lesson to be learned here is simply that the non-adaptive immune status of immunodeprived mice can significantly influence the therapeutic evaluation of antibody-based drugs like HB2-SAPORIN and give a misleading overestimate of their intrinsic anti-tumour potency. On the positive side such in vivo animal models also provide a means of delineating and defining contributory additivities between mechanistically different antibody-mediated anti-tumour activities and provide us with valuable insights that may eventually assist us in the development of multi-modal targeted approaches for the treatment of human malignancies.

### ACKNOWLEDGEMENTS

This work was supported by the children's leukaemia research charity Leukaemia Busters. We would like to thank Dr Finbarr Cotter for generously providing the NOD/SCID mice breeding pairs and the staff of the Biomedical Research Facility (University of Southampton) for their expert technical assistance.

### REFERENCES

Adams RA, Pothier L, Flowers A, Lazarus H, Farber S and Foley GE (1970) The question of stemlines in human acute leukemia. Comparison of cells isolated *in vitro* and *in vivo* from a patient with acute lymphoblastic leukemia. *Experimental Cell Research* **62**: 5–10

Baxter AG and Cooke A (1993) Complement lytic activity has no role in the pathogenesis of autoimmune diabetes in NOD mice. *Diabetes* **42**: 1574–1578

Bhatia M, Wang JCY, Kapp U, Bonnet D and Dick JE (1997) Purification of primitive human hematopoietic cells capable of repopulating immune-deficient mice. *PNAS* **94**: 5320–5325

Bosma GC, Custer RP and Bosma MJ (1983) A severe combined immunodeficiency mutation in the mouse. *Nature* **301**: 527–530

Cesano A, O'Connor R, Lange B, Finan J, Rovera G and Santoli D (1991) Homing and progression patterns of childhood acute lymphoblastic leukaemias in severe combined immunodeficient mice. *Blood* **77**: 2463–2474

Christianson SW, Shultz LD and Leiter EH (1993) Adoptive transfer of diabetes into immunodeficient NOD-scid/scid mice. Relative contributions of CD4+ and CD8+ T-cells from diabetic versus prediabetic NOD. NON-Thy-1a donors. *Diabetes* **42**: 44–55

Cikes M, Friberg S and Klein G (1973) Progressive loss of H-2 antigens with concomitant increase of cell-curface antigen(s) determined by Moloney Leukemia Virus in cultured murine lymphomas. *Journal of National Cancer Instute* **50**: 347–362

Custer RP, Bosma GC and Bosma MJ (1985) Severe combined immunodeficiency (SCID) in the mouse. Pathology, reconstitution, neoplasms. *Am J Pathol* **120**: 464–477

Dick JE (1996) Normal and leukemic human stem cells assayed in SCID mice. *Semin Immunol*, **8**: 197–206

Dorshkind K, Keller GM, Phillips RA, Miller RG, Bosma GC, O'Toole M and Bosma MJ (1984) Functional status of cells from lymphoid and myeloid tissues in mice with severe combined immunodeficiency disease. *J Immunol* **132**: 1804–1808

Dorshkind K, Pollack SB, Bosma MJ and Phillips RA (1985) Natural killer cells are present in mice with severe combined immunodeficiency. *Journal of Immunology* **134**: 3798–3801

Flavell DJ (1996) Modelling human leukaemia and lymphoma in severe combined immunodeficient (Scid) mice: practical applications. *Haematological Oncology* **14**: 67–82

Flavell DJ, Boehm DA, Noss A and Flavell SU (1997) Comparison of the potency and therapeutic efficacy of the anti-CD7 immunotoxin HB2-Saporin constructed with one or two saporin moieties per immunotoxin molecule. *British Journal of Cancer* **75**: 1035–1043

Flavell DJ, Warnes S, Noss A and Flavell SU (1998) Host-mediated antibody dependent cellular cytotoxicity (ADCC) contributes to the *in vivo* therapeutic efficacy of an anti-CD7-saporin immunotoxin in a SCID mouse model of human T-ALL. *Cancer Research* **58**: 5787–5794

Kamel-Reid S, Letarte M, Sirard C, Doedens M, Grunberger T, Fulop G, Freedman MH, Phillips RA and Dick JE (1989) A model of human acute lymphoblastic leukemia in immune-deficient SCID mice. *Science* **246**: 1597–1600

Kamel-Reid S, Letarte M, Doedens M, Greaves A, Murdoch B, Grunberger T, Lapidot T, Thorner P, Freedman MH, Phillips RA and Dick JE (1991) Bone marrow from children in relapse with pre-B acute lymphoblastic leukaemia proliferates and disseminates rapidly in SCID mice. *Blood* **78**: 2973–2981

Kataoka S, Satoh J, Fujiya H, Toyota T, Suzuki R, Itoh K and Kumagai K (1983) Immunologic aspects of the nonobese diabetic (NOD) mouse. Abnormalities of cellular immunity. *Diabetes* **32**: 247–253

Kollmann TR, Kim A, Zhuang X, Hachamovitch M and Goldstein H (1994) Reconstitution of SCID mice with human lymphoid and myeloid cells after transplantation with human fetal bone marrow without the requirement for exogenous human cytokines. *PNAS* **91**: 8032–8036

Kotloff DB, Bosma MJ and Ruetsch NR (1993) Scid mouse pre-B cells with intracellular μ chains: analysis of recombinase activity and IgH gene rearrangements. *Int Immunol* **5**: 383–391

Lapidot T, Pflumio F, Doedens M, Murdoch B, Williams DE and Dick JE (1992) Cytokine stimulation of multilineage hematopoiesis from immature human cells engrafted in SCID mice. *Science* **255**: 1137–1141

© 2000 Cancer Research Campaign

Morland BJ, Barley J, Boehm D, Flavell SU, Ghaleb N, Kohler JA, Okayama K, Wilkins B and Flavell DJ (1994) Effectiveness of HB2(anti-CD7)-saporin immunotoxin in an *in vivo* model of human T-cell leukaemia developed in severe combined immunodeficient mice. *British Journal of Cancer* **69**: 279–285

Pilarski LM, Hipperson G, Seeberger K, Pruski E, Coupland RW and Belch AR (2000) Myeloma progenitors in the blood of patients with aggressive or minimal disease: engraftment and self-renewal of primary human myeloma in the bone marrow of NOD SCID mice. *Blood* **95**: 1056–1065

Prochazka M, Gaskins HR, Shultz LD and Leiter EH (1992) The non-obese diabetic scid mouse:model for spontaneous thymomagenesis associated with immunodeficiency. *PNAS* **89**: 3290–3294

Serreze DV and Leiter EH (1988) Defective activation of T suppressor cell function in nonobese diabetic mice. Potential relation to cytokine deficiencies. *J Immunol* **140**: 3801–3807

Serreze DV, Gaskins HR and Leiter EH (1993) Defects in the differentiation and function of antigen presenting cells in NOD/Lt mice. *J Immunol* **150**: 2534–2543

Shpitz B, Fernandes BJ, Mullen JBM, Roder JC and Gallinger S (1994) Improved engraftment of human tumours in SCID mice pretreated with radiation and anti-asialo GMI. *Anticancer Research* **14**: 1927–1934

Shultz L, Schweitzer PA, Christianson SW, Gott B, Schweitzer IB, Tennent B, McKenna S, Mobraaten L, Rajan TV, Greiner DL and Leiter EH (1995) Multiple defects in innate and adaptive immunologic function in NOD/LtSz-scid mice. *The Journal of Immunology* **154**: 180–191

Thorpe PE, Brown ANF, Jr, JAGB, Foxwell BMJ and Stirpe F (1985) An immunotoxin composed of monoclonal anti-Thy 1.1 antibody and a ribosome-inactivating protein from *Saponaria officinalis*: potent antitumor effects in vitro and in vivo. *Journal of National Cancer Institute* **75**: 151–159

Wang JCY, Lapidot T, Cashman JD, Sirard C, Doedens M, Laraya P, Addy L, Minden M, Keating A, Eaves AC, Eaves CJ and Dick JE (1995) Engraftment of primitive normal and leukemic hematopoetic cells from chronic phase CML patients in immunodeficient mice. *Blood* **86**: 601a

Workman P, Twentyman P, Balkwill F, Balmain A, Chaplin D, Double J, Embleton J, Newell D, Raymond R, Stables J, Stephens T and Wallace J (1998) United Kingdom Co-ordinating Committee on Cancer Research (UKCCCR) guidelines for the welfare of animals in experimental neoplasia (second edition). *Br J Cancer* **77**: 1–10

Yu J, Dialynas DP, Shao LE, Lee MJ, Gold DP and Yu AL (1999) Fetal cord blood facilitates engraftment of primary childhood T-cell acute lymphoblastic leukaemia in immunodeficient mice and enhances leukaemia colony formation in vitro. *Proc Am Assoc Cancer Res* **40**: 660 (abstract #4352)

*© 2000 Cancer Research Campaign*

## References

[PDF_00504] Adams R. A., Pothier L., Flowers A., Lazarus H., Farber S., Foley G. E. (1970). The question of stemlines in human acute leukemia. Comparison of cells isolated in vitro and in vivo from a patient with acute lymphoblastic leukemia.. Exp Cell Res.

[PDF_00508] Baxter A. G., Cooke A. (1993). Complement lytic activity has no role in the pathogenesis of autoimmune diabetes in NOD mice.. Diabetes.

[PDF_00511] Bhatia M., Wang J. C., Kapp U., Bonnet D., Dick J. E. (1997). Purification of primitive human hematopoietic cells capable of repopulating immune-deficient mice.. Proc Natl Acad Sci U S A.

[PDF_00514] Bosma G. C., Custer R. P., Bosma M. J. (1983). A severe combined immunodeficiency mutation in the mouse.. Nature.

[PDF_00516] Cesano A., O'Connor R., Lange B., Finan J., Rovera G., Santoli D. (1991). Homing and progression patterns of childhood acute lymphoblastic leukemias in severe combined immunodeficiency mice.. Blood.

[PDF_00519] Christianson S. W., Shultz L. D., Leiter E. H. (1993). Adoptive transfer of diabetes into immunodeficient NOD-scid/scid mice. Relative contributions of CD4+ and CD8+ T-cells from diabetic versus prediabetic NOD.NON-Thy-1a donors.. Diabetes.

[PDF_00523] Cikes M., Friberg S., Klein G. (1973). Progressive loss of H-2 antigens with concomitant increase of cell-surface antigen(s) determined by Moloney leukemia virus in cultured murine lymphomas.. J Natl Cancer Inst.

[PDF_00527] Custer R. P., Bosma G. C., Bosma M. J. (1985). Severe combined immunodeficiency (SCID) in the mouse. Pathology, reconstitution, neoplasms.. Am J Pathol.

[PDF_00530] Dick J. E. (1996). Normal and leukemic human stem cells assayed in SCID mice.. Semin Immunol.

[PDF_00532] Dorshkind K., Keller G. M., Phillips R. A., Miller R. G., Bosma G. C., O'Toole M., Bosma M. J. (1984). Functional status of cells from lymphoid and myeloid tissues in mice with severe combined immunodeficiency disease.. J Immunol.

[PDF_00536] Dorshkind K., Pollack S. B., Bosma M. J., Phillips R. A. (1985). Natural killer (NK) cells are present in mice with severe combined immunodeficiency (scid).. J Immunol.

[PDF_00542] Flavell D. J., Boehm D. A., Noss A., Flavell S. U. (1997). Comparison of the potency and therapeutic efficacy of the anti-CD7 immunotoxin HB2-saporin constructed with one or two saporin moieties per immunotoxin molecule.. Br J Cancer.

[PDF_00539] Flavell D. J. (1996). Modelling human leukemia and lymphoma in severe combined immunodeficient (SCID) mice: practical applications.. Hematol Oncol.

[PDF_00546] Flavell D. J., Warnes S., Noss A., Flavell S. U. (1998). Host-mediated antibody-dependent cellular cytotoxicity contributes to the in vivo therapeutic efficacy of an anti-CD7-saporin immunotoxin in a severe combined immunodeficient mouse model of human T-cell acute lymphoblastic leukemia.. Cancer Res.

[PDF_00553] Kamel-Reid S., Letarte M., Doedens M., Greaves A., Murdoch B., Grunberger T., Lapidot T., Thorner P., Freedman M. H., Phillips R. A. (1991). Bone marrow from children in relapse with pre-B acute lymphoblastic leukemia proliferates and disseminates rapidly in scid mice.. Blood.

[PDF_00550] Kamel-Reid S., Letarte M., Sirard C., Doedens M., Grunberger T., Fulop G., Freedman M. H., Phillips R. A., Dick J. E. (1989). A model of human acute lymphoblastic leukemia in immune-deficient SCID mice.. Science.

[PDF_00558] Kataoka S., Satoh J., Fujiya H., Toyota T., Suzuki R., Itoh K., Kumagai K. (1983). Immunologic aspects of the nonobese diabetic (NOD) mouse. Abnormalities of cellular immunity.. Diabetes.

[PDF_00561] Kollmann T. R., Kim A., Zhuang X., Hachamovitch M., Goldstein H. (1994). Reconstitution of SCID mice with human lymphoid and myeloid cells after transplantation with human fetal bone marrow without the requirement for exogenous human cytokines.. Proc Natl Acad Sci U S A.

[PDF_00565] Kotloff D. B., Bosma M. J., Ruetsch N. R. (1993). Scid mouse Pre-B cells with intracellular mu chains: analysis of recombinase activity and IgH gene rearrangements.. Int Immunol.

[PDF_00568] Lapidot T., Pflumio F., Doedens M., Murdoch B., Williams D. E., Dick J. E. (1992). Cytokine stimulation of multilineage hematopoiesis from immature human cells engrafted in SCID mice.. Science.

[PDF_00573] Morland B. J., Barley J., Boehm D., Flavell S. U., Ghaleb N., Kohler J. A., Okayama K., Wilkins B., Flavell D. J. (1994). Effectiveness of HB2 (anti-CD7)--saporin immunotoxin in an in vivo model of human T-cell leukaemia developed in severe combined immunodeficient mice.. Br J Cancer.

[PDF_00577] Pilarski L. M., Hipperson G., Seeberger K., Pruski E., Coupland R. W., Belch A. R. (2000). Myeloma progenitors in the blood of patients with aggressive or minimal disease: engraftment and self-renewal of primary human myeloma in the bone marrow of NOD SCID mice.. Blood.

[PDF_00581] Prochazka M., Gaskins H. R., Shultz L. D., Leiter E. H. (1992). The nonobese diabetic scid mouse: model for spontaneous thymomagenesis associated with immunodeficiency.. Proc Natl Acad Sci U S A.

[PDF_00587] Serreze D. V., Gaskins H. R., Leiter E. H. (1993). Defects in the differentiation and function of antigen presenting cells in NOD/Lt mice.. J Immunol.

[PDF_00584] Serreze D. V., Leiter E. H. (1988). Defective activation of T suppressor cell function in nonobese diabetic mice. Potential relation to cytokine deficiencies.. J Immunol.

[PDF_00589] Shpitz B., Fernandes B. J., Mullen J. B., Roder J. C., Gallinger S. (1994). Improved engraftment of human tumours in SCID mice pretreated with radiation and anti-asialo GM1.. Anticancer Res.

[PDF_00592] Shultz L. D., Schweitzer P. A., Christianson S. W., Gott B., Schweitzer I. B., Tennent B., McKenna S., Mobraaten L., Rajan T. V., Greiner D. L. (1995). Multiple defects in innate and adaptive immunologic function in NOD/LtSz-scid mice.. J Immunol.

[PDF_00596] Thorpe P. E., Brown A. N., Bremner J. A., Foxwell B. M., Stirpe F. (1985). An immunotoxin composed of monoclonal anti-Thy 1.1 antibody and a ribosome-inactivating protein from Saponaria officinalis: potent antitumor effects in vitro and in vivo.. J Natl Cancer Inst.

